# Utilization of Western Medicine and Traditional Chinese Medicine Services by Physicians and Their Relatives: The Role of Training Background

**DOI:** 10.1093/ecam/nep094

**Published:** 2011-03-13

**Authors:** Nicole Huang, Yiing-Jenq Chou, Long-Shen Chen, Cheng-Hua Lee, Pen-Jen Wang, Jen-Huoy Tsay

**Affiliations:** ^1^Institute of Hospital and Health Care Administration, School of Medicine, National Yang Ming University, Taipei 112, Taiwan; ^2^Institute of Public Health & Department of Public Health, School of Medicine, National Yang Ming University, Taipei 112, Taiwan; ^3^Taiwan's Bureau of National Health Insurance, College of Social Science, National Taiwan University, Taipei, Taiwan; ^4^Department of Social Work, College of Social Science, National Taiwan University, Taipei, Taiwan

## Abstract

Despite extensive efforts to improve the attitude and practice of physicians with respect to complementary and alternative medicine (CAM), the role of training background on physician's own utilization of mainstream Western medicine (WM) and CAM remains unclear. We aimed to compare personal utilizations of WM and traditional Chinese medicine (TCM) among doctors trained in WM only, TCM only or both. A retrospective population-based study was conducted using the 2004 Taiwan's National Health Insurance data. A total of 103 879 doctors and their relatives and 2 623 658 other adults with equivalent socioeconomic status were analyzed. Ambulatory care utilization of WM and TCM services was compared using the following three measures: probability of any use, number of visits and total annual expenditure. Doctors who were trained in Western medicine only (WMDs) had the highest WM use, followed by doctors who were trained in both (WMD-CMDs), while Chinese medicine-trained doctors (CMDs) had the lowest use. For TCM use, a reverse pattern was observed. Similar patterns were found among doctors' relatives. Compared with other adults with equivalent socioeconomic status, both the CMDs and WMD-CMDs had a greater use of TCM services. For WM, although the WMDs' probability and frequency of usage were similar to other adults, they incurred considerably higher expenditure. The use of WM and TCM by doctors and their relatives was significantly associated with the training background of the doctors. This highlights the importance of how increasing knowledge and understanding of other medical discipline may influence a practitioner's care-providing behaviors.

## 1. Introduction

Complementary and alternative medicine (CAM) services have undergone a surge of increased public popularity and physicians play a significant role in facilitating better integration between CAM and Western biomedicine. Many believe that physicians' personal experiences may strongly influence their beliefs in and practice of CAM [1–4]. However, only limited empirical evidence is available on personal utilization of CAM by physicians. More importantly, a growing number of physicians have expressed interests in studying CAM [5]. In some countries, more physicians are being trained in both disciplines. The behaviors of this physician group may be of great interest to the public and medical community. Very few studies have examined the use of Western Medicine (WM) and CAM ambulatory services by physicians either personally, or by their family. Moreover, none has investigated whether their utilization of these two sources of care varies with respect to their training background in WM and CAM. Such findings may help us to discover whether variation in knowledge of WM or CAM leads to a difference in their use of different sources of care.

Although the literature on Western medicine-trained doctors' (WMDs) knowledge of, attitudes toward, and practice behaviors regarding CAM is extensive [1–4, 6–19], there is scarce information published on personal experiences with CAM therapy. The source of care used by WMDs or their family members may reflect their underlying attitude toward CAM. The limited evidence available in the literature shows that the reported personal use ranges from as low as 9% in UK to as high as 90% in China, and that this is most likely due to cultural norms, regional differences and sampling methods [9, 10, 14, 20, 21]. More rigorous studies using national and representative samples are required.

Furthermore, since CAM is becoming popular, CAM practitioners may have an increasingly strong influence on their patients. Hence, understanding their personal health-seeking behavior is equally important. However, few studies have paid some amount of attention to the health services utilization patterns of CAM professionals. Only one recent Korean study has investigated differences in attitude, understanding and practice experience with alternative medicine modalities among doctors who follow Oriental practices and doctors who follow WM practices [9]. However, no information is available on their personal utilizations of WM or CAM services.

Of the different CAM therapies, traditional Chinese medicine (TCM) is a well-recognized CAM modality. In addition to recent interest in TCM in Western societies, TCM is widely practiced in Oriental societies such as China, Taiwan, Japan, Korea, Singapore and Vietnam for more than 2000 years [22–24]. Since the National Health Insurance (NHI) Program was implemented in Taiwan in 1995, TCM has been an integral part of its universal and comprehensive insurance coverage. Both WM and TCM-trained doctors (CMDs) co-exist in Taiwan's health care system. In addition, Taiwan allows some doctors who have passed official licensure examinations, to own dual licenses in Taiwan. This feature of Taiwan's medical system allows us to compare personal and family utilizations of health care by dual-trained physicians with those by physicians trained only in either WM or TCM under the NHI program.

## 2. Methods

### 2.1. Background: Training of WMD and CMD in Taiwan

In Taiwan, a certified WMD needs to be formally educated in a medical school, successfully complete training requirements and pass a national license examination (the Physician License Examination, PLE). On the other hand, no formal medical school education is required to be a licensed traditional CMD. First, similar to WMDs, students who obtain a formal medical degree in Chinese medicine from medical school and pass a national license examination (the Chinese Medicine Physician License Examination, CMPLE) can then be certified as a CMD [23]. Alternatively, for historical reasons, individuals without formal medical training can also be certified through a two-stage special license examination (the Chinese Medicine Qualifying Examination and the Chinese Medicine Special Examination) [23]. However, in order to assure the quality of TCM care and improve the credentials of Chinese Medicine practitioners, the two-stage special license examinations for people without formal medical school training will be terminated in 2011. Furthermore, some doctors may have dual licenses in Taiwan. First, the Chinese medicine program graduates, who entered the program before 1995 and passed the CMPLE, are also eligible to take the PLE to be certified as a WMD. Second, before a policy change in December 2007, certified WMDs with a strong interest in TCM and who have taken required Chinese medicine courses are eligible for the CMPLE to become a licensed CMD.

### 2.2. Data Sources and Study Population

The 2004 NHI Enrollment File, the NHI Ambulatory Care Claims Data, the NHI Major Disease File, the Medical Personnel Registry from the Department of Health, the hospital/clinic registry, the household registry and death certificates were used in this study. This population-based cross-sectional study includes a total of 13 652 794 adults (aged ≥25 years) who lived in a non-remote area and who were fully enrolled in the NHI program in 2004.

The study subjects were categorized into seven groups: WMDs, relatives of WMDs, WMD-CMDs, relatives of WMD-CMDs, CMDs, relatives of CMDs and non-health professional adults from similar socioeconomic background. The identification number and date of birth of individuals were matched against the 2004 Medical Personnel Registry to identify those individuals with a recorded license to practice as a WMD or as a CMD, or both.

Next, an individual living in the same household as a doctor was considered the doctor's family member, regardless of his/her exact relationship with that doctor. For this, we used the NHI Medical Personnel Registry and the household registry. A household was defined as a person or group of people registered in the same dwelling. Those individuals who were neither doctors nor living in the same household as a doctor, were defined as other adults (the comparison group).

The health status and the socioeconomic status (SES) of doctors and their family members may be relatively better than that of other adults. Therefore, in order to make doctors, their relatives and the comparison group more comparable, this study included only individuals (physicians, relatives and other adults) who did not have any listed major disease (13 146 866). The listed major diseases are an official disease list used by the NHI program to identify individuals with major diseases or injuries. In Taiwan, people with specific major diseases or injury diagnoses from medical doctors can apply for a “major disease/injury card”. These cardholders are exempted from the cost sharing required under the NHI program. On the basis of the Injury Severity Index, the NHI major disease list includes 30 major disease or injury types such as cancer, end-stage renal disease, chronic psychotic disorder, cirrhosis of liver and acquired immunodeficiency syndrome. We obtained the information on individual's major disease status from the NHI Major Diseases file. We used the ownership of a major disease/injury card to represent the subject's health status. Sensitivity analyses were conducted by including individuals with major diseases or injuries and using total population as the comparison group. The results remain robust.

**Figure 1:**
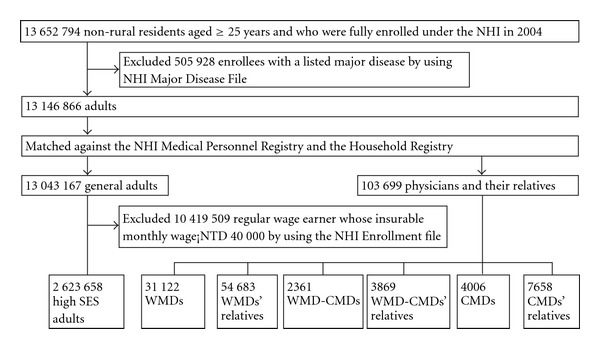
Data and the sampling process.

Furthermore, to assure better comparability, we included only those who were regular wage earners such as civil servants, government employees, private sector employees, teachers, employers and professionals, who had an insurable monthly wage ≥40 000 NTD, in our comparison group (other high SES adults). Sensitivity analyses were also conducted using different cut-off points (NTD 20 000 and no minimum cutoff) and the results remained stable. The final fixed cohort included 2 727 537 individuals. There were 31 122 WMDs, 54 863 relatives of WMDs, 4006 CMDs, 7658 relatives of CMDs, 2361 doctors with dual licenses (WMD-CMD), 3869 relatives of WMD-CMDs and 2 623 658 high SES adults. Data and the sampling process are shown in Figure 1.

### 2.3. Dependent Variables

#### 2.3.1. TCM Use

TCM use in a year under the NHI program was measured in three ways: probability of using any of the TCM ambulatory services, number of visits to a CMD and the total expenditure on TCM. The information on TCM use was obtained from the NHI claims data. Commonly practiced TCM modalities such as acupuncture and moxibustion, manual therapy and Chinese herbal remedies are covered by the NHI program.

#### 2.3.2. WM Use

WM use in a year under the NHI program was measured in three ways: probability of using any of the WM ambulatory services, number of visits to a WMD and the total annual expenditure on WM.

### 2.4. Statistical Analysis

The unit of observation in this study was the person-year. A set of three variables (age, gender and geographic location) was used to adjust for differences in health care utilization across each subgroup. The distribution of each variable across the subgroups is shown in Table 1. The probability of using TCM services in 1 year was constructed as a binary variable (0 = no visit to a CMD, 1 = at least one visit to a CMD) and was estimated using logistic regression. The number of TCM visits and the expenditure were modeled using two-part models [25, 26]. In the first part, the probability of at least one visit to a CMD was estimated using logistic regression. The number of visits to a CMD, and the TCM expenditure, conditional on a positive TCM use, were then estimated using ordinary least square (OLS) linear regression. Since the distribution of TCM expenditure was highly skewed, the natural logarithm of TCM expenditure was used in the models. The predicted log medical expenditure was re-transformed to a raw scale in order to calculate the predicted total medical expenditure using the smearing technique [25]. Similar analyses were applied to WM use. A significance level of *α* = 0.05 and a power of 0.80 were used. All analyses were conducted using the statistical packages SAS 8.2 and STATA 8. The data linkage process was conducted within the Bureau of NHI and followed the government's confidentiality regulations during the linkage and analysis processes.

## 3. Results

### 3.1. Sample Description

The basic demographic characteristics of the seven subgroups are presented in Table 1. Male practitioners dominated all three types of doctors by a substantial margin. Of all seven groups, relatives of WMDs had the highest mean age (50.8 years). WMD-CMDs were younger than the high SES adults and had the lowest mean age (41.3 years). Almost 40% of WMDs and their relatives were located in the Taipei region, which was similar to the results for high SES adults. On the other hand, over 30% of WMD-CMDs, CMDs and their relatives were located in the central region of Taiwan.

### 3.2. WM Use

Adjusted measures of TCM and WM use among the seven subgroups are presented in Table 2. After adjusting for age, gender and geographic location, a dose-response relationship by training background of the doctors was evident. WMDs (84%) not only had a substantially higher probability of using WM in a year compared with CMDs (57%), but also showed a slightly higher use than those with both WM and Chinese medicine licenses (81%). WMDs had the highest number of WM visits per year (10.91), followed by WMD-CMDs (9.41) and finally CMDs (3.91). Similarly, the expenditure of WMDs (NTD 13 415/year) was 1.6 times higher than that of WMD-CMDs (NTD 8627/year) and 3.5 times higher than that of CMDs (NTD 3852/year). A similar dose-response relationship was also observed in WM use by the physicians' relatives.

### 3.3. TCM Use

After adjustment, of the three doctor subgroups, CMDs were most likely to use TCM services, had the most visits and the highest total of TCM expenditure. They were followed by WMD-CMDs, and WMDs had the lowest TCM usage. However, the magnitude of differences in TCM use among different doctor subgroups was much larger than that for WM use. For example, CMDs had 17.6 more TCM visits and incurred 71.3 times higher TCM expenditure in 1 year than WMDs, while WMDs had only seven more WM visits and incurred 3.48 times higher WM expenditure than CMDs.

For their relatives, a similar pattern was observed. CMDs' relatives had the highest levels of TCM use of all three family groups, but the difference in TCM utilizations observed between relatives of CMDs and WMDs were not as large as between the doctors themselves. In summary, there was a clear gradient in the use of TCM across the subgroups. CMDs and their relatives had the highest TCM use, then WMD-CMDs and their relatives, and finally, WMDs and their relatives had the lowest TCM use.

One additional pattern is worth noting. In general, relatives of doctors tended to have higher health care utilizations, either WM or TCM services, than the doctors themselves. The only exception was that CMDs were more likely to use TCM in 1 year (70%), had more frequent TCM visits (17.87 visits/year) and had higher TCM expenditure (NTD 9557/year) than their relatives (59%, 9.16 visits/year, NTD 5014/year, resp.).

### 3.4. Comparison with the Rates for High SES Adults

After controlling for age, gender and geographic location, high SES adults were reported to have similar probability of WM use and frequency of visits as WMDs. However, their total WM expenditure was considerably lower than that of WMDs. In addition, their WM use was substantially lower than that of the relatives of doctors who had been trained in WM. Similarly, in terms of TCM services, doctors who had been trained in TCM along with their relatives had made more frequent visits and higher total TCM expenditure than high SES adults. Furthermore, doctors who had only been trained in WM along with their relatives had less frequent TCM visits and lower TCM expenditure than high SES adults.

## 4. Discussion

To our knowledge, this is the first population-based study to investigate how the type of medical training they received may influence doctors' and their relatives' usage of WM and TCM services. In addition, we compared these findings with the usage of high SES adults. The findings indicate that doctors with different training backgrounds do differ significantly in their use of WM and TCM services as covered by the NHI program in Taiwan. Doctors predominantly sought care offered by their own medical discipline. Their utilization of services offered by the other discipline was even lower than those of high SES adults. Overall, 99% of the WMDs' total ambulatory care expenditure that was incurred was on WM care and this contrasts with 71% of the total expenditure incurred by CMDs using TCM services. Those with training in both WM and TCM had a more balanced mix of WM and TCM service use. There are some plausible explanations. First, different levels of WM and/or TCM knowledge among the physicians with different training backgrounds might contribute to the significant differences in their care-seeking behaviors [27–29]. Greater knowledge of one discipline may lead to a higher confidence and better assessment of strengths and advantages of that particular discipline. This may significantly affect their choice of treatments.

Second, doctors may have easier access to care offered by practitioners of their own medical disciplines due to familiarity with their own medical system and proximity to services. Finally, in addition to these two factors, barriers such as competition, traditional distrust or hostility, may impede doctors to seek care from practitioners of the other discipline. In some instances, particularly for minor ailments, WM and TCM can substitute for one another [9, 30]. As TCM is legally institutionalized and many of its services are covered by the NHI program, TCM is in competition with WM in some situations in Taiwan. This competitive position may cause WMDs and CMDs to adhere to their own medical discipline as a way to differentiate themselves from each other and may lead them to rigidly exclude the possibility of using suitable care offered by the other system. These reasons may not apply to physicians trained in both WM and TCM, as shown by the fact that their use of services offered by the other system was higher than that of doctors trained in only one discipline.

According to the literature [1, 3, 6, 9, 10, 24, 31], the socialization process during medical education/training may play a significant role in shaping a doctor's views and practices. The case of WMD-CMDs in Taiwan serves as an interesting illustration. As many medical schools are WM-oriented, the basic principles and the recent developments of TCM therapies are often overlooked in the training of WMDs. This explicitly limits the exposure of future WMDs to TCM and provides no opportunity to rectify many incorrect stereotypes or myths about TCM. Similarly, since many practicing CMDs learned TCM through self-study or an apprenticeship, the same socialization problems occur in the training of CMDs. In contrast, the training of WMD-CMDs encourages better understanding of both disciplines through courses, role models and internships and this helps to create a positive environment for integration of the two disciplines.

### 4.1. Use of WM and TCM by Doctors, Doctors' Relatives and High SES Adults

Another interesting finding is the use of WM and TCM by doctors' relatives. First, the preferences and utilizations of TCM and WM services among the family members of a doctor follow the preferences of the doctor. Our findings suggest that the training background of doctors may influence how their relatives use specific sources of care. Second, in general, family members of doctors not only make greater use of services offered by the discipline of the doctor in the family, but also those offered by the other discipline, than the doctors themselves. Some barriers, which hinder physicians from seeking care, such as their denial of vulnerability, their concerns about information confidentiality, their resistance to entering the patient role, their embarrassment of showing their weaknesses to colleagues from their own or another system and their scheduling difficulties, may not apply to their family members [32–35]. The one exception was a higher TCM use among CMDs than their relatives. A possible explanation may be that the TCM's humanistic, holistic philosophy and whole person management may help to mitigate some barriers described above. Future large-scale health professional surveys may help to offer more concrete explanations.

Furthermore, when doctors and other high SES adults were compared, some interesting patterns were noted. First, doctors and their relatives, presumably a group of medically better-informed consumers, consumed more ambulatory care resources than other high SES adults. For TCM services, either CMDs, WMD-CMDs or their relatives, who have greater knowledge and better access to TCM services, had a greater use of TCM services than other high SES adults. For WM, although WMDs' probability and frequency of usage were similar to high SES adults', WMDs incurred considerably higher expenditure than the comparison group (high SES adults). The differences observed between high SES adults and relatives of doctors who were trained in WM were even more evident. The findings are consistent with findings in previous studies regarding physicians as patients [27, 33]. Moreover, it is likely that the present study may have underestimated actual service utilizations by physicians. In addition to the formal venue of care, physicians may obtain medical advice or treatment informally through self-treatment or courtesy consultations, which most patients do not have access to.

A few limitations of this study should be noted. First, this study might suffer from certain inherent limitations due to the use of administrative and claims data that lack information on the decision process and the reasons why doctors, their relatives and people in general choose WM or TCM. Second, misclassification may be a source of bias. Those who lived with two or more different types of doctors were classified in the order of WMD, WMD-CMD and CMD. Sensitivity analyses were conducted on different assigning algorithms and the results remained robust. Furthermore, since the household registry can only link individuals co-residing with physicians, family members of physicians who lived in a different household may be classified under another group. Third, as noted above, the reported TCM utilization rates across all groups in this study may be underestimated since the study focused only on WM and TCM services covered by the NHI program in Taiwan. For example, physicians or people in Taiwan may purchase herbal remedies through alternative route other than the NHI program. Finally, our study only focused on physicians' personal and family uses of CAM. From a population perspective, it may be more important in future research to investigate physicians' practice regarding CAM.

Nevertheless, this study is a first attempt to compare personal and family use of WM and TCM services by doctors with different training backgrounds. The findings indicate a strong preference for seeking the care offered by their own discipline. Less extreme preferences of WM or TCM care were observed among doctors who were trained in both disciplines. This highlights the importance of how increasing the knowledge and the understanding of both medical disciplines may influence a practitioner's care-seeking behavior. More importantly, it may help to improve the integration of WM and TCM in their practice, since physicians' personal experiences may strongly influence their beliefs and their practices.

## Figures and Tables

**Table 1:** Demographic characteristics of WMDs, CMDs, dual-trained doctors, doctor's relatives and the high SES adults.

	WMDs (*n* = 31 122)	Relatives of WMD (*n* = 54 863)	WMD-CMDs (*n* = 2361)	Relatives of WMD-CMD (*n* = 3869)	CMDs (*n* = 4006)	Relatives of CMD (*n* = 7658)	High SES adults (*n* = 2 623 658)
	*n* (%)	*n* (%)	*n* (%)	*n* (%)	*n* (%)	*n* (%)	*n* (%)
Age (years, mean ± SD)	46.1 ± 14.9	50.8 ± 17.7	41.3 ± 8.0	47.4 ± 16.0	47.0 ± 11.5	46.8 ± 16.2	44.9 ± 12.9
Gender							
Male	27 576 (88.6)	22 899 (41.7)	2029 (85.9)	1342 (34.7)	3139 (78.4)	3096 (40.4)	1 559 179 (59.4)
Female	3546 (11.4)	31 964 (58.3)	332 (14.1)	2527 (65.3)	867 (21.6)	4562 (59.6)	1 064 479 (40.6)
Geographic location							
Taipei branch	12 087 (38.8)	21 106 (38.5)	571 (24.2)	965 (24.9)	1202 (30.0)	2145 (28.0)	1 073 316 (40.9)
Northern branch	2926 (9.4)	5522 (10.1)	182 (7.7)	323 (8.3)	448 (11.2)	968 (12.6)	420 534 (16.0)
Central branch	5678 (18.2)	9937 (18.1)	941 (39.9)	1420 (36.7)	1222 (30.5)	2391 (31.2)	394 943 (15.1)
South branch	4351 (14.0)	9290 (16.9)	393 (16.6)	716 (18.5)	527 (13.2)	1066 (13.9)	295 914 (11.3)
Kao-Ping branch	5541 (17.8)	8099 (14.8)	231 (9.8)	400 (10.3)	552 (13.8)	996 (13.0)	395 624 (15.1)
East branch	539 (1.7)	909 (1.7)	43 (1.8)	45 (1.2)	55 (1.4)	92 (1.2)	43 327 (1.7)

WMDs: Western Medicine-Trained Doctors; CMDs: Chinese Medicine-Trained Doctors; SES: Socioeconomic status.

**Table 2:** Adjusted utilization measures of WMDs, CMDs, dual-trained doctors, doctor's relatives and the high SES adults.

	WMDs (*n* = 31 122)	Relatives of WMD (*n* = 54 863)	WMD-CMDs (*n* = 2361)	Relatives of WMD-CMDs (*n* = 3869)	CMDs (*n* = 4006)	Relatives of CMDs (*n* = 7658)	High SES adults (*n* = 2 623 658)
	Mean (SE)	Mean (SE)	Mean (SE)	Mean (SE)	Mean (SE)	Mean (SE)	Mean (SE)
WM							
Probability of use	0.84 (0.00)	0.89 (0.00)	0.81 (0.00)	0.90 (0.00)	0.57 (0.00)	0.81 (0.00)	0.89 (0.00)
Visits	10.91 (0.04)	16.02 (0.04)	9.41 (0.05)	14.71 (0.14)	3.91 (0.04)	9.70 (0.07)	11.01 (0.00)
Expenditures (in NTD)	13 415 (79)	17 186 (71)	8627 (58)	14 797 (224)	3852 (66)	9092 (110)	9531 (31)
TCM							
Probability of use	0.06 (0.00)	0.22 (0.00)	0.22 (0.00)	0.31 (0.00)	0.70 (0.00)	0.59 (0.00)	0.31 (0.00)
Visits	0.24 (0.00)	1.13 (0.00)	2.07 (0.02)	2.40 (0.01)	17.87 (0.06)	9.16 (0.03)	1.67 (0.00)
Expenditures (in NTD)	134 (0)	624 (1)	1133 (8)	1324 (7)	9557 (29)	5014 (15)	915 (0)

A set of three variables (age, gender, and geographic location) was used to adjust for differences in health care utilization across each subgroup.
